# Defining data-driven primary transcript annotations with *primaryTranscriptAnnotation* in R

**DOI:** 10.1093/bioinformatics/btaa011

**Published:** 2020-01-09

**Authors:** Warren D Anderson, Fabiana M Duarte, Mete Civelek, Michael J Guertin

**Affiliations:** b1 Center for Public Health Genomics, University of Virginia, Charlottesville, VA 22908, USA; b2 Department of Stem Cell and Regenerative Biology, Harvard University, Cambridge, MA 02138, USA; b3 Department of Biomedical Engineering, University of Virginia, Charlottesville, VA 22903, USA; b4 Department of Biochemistry and Molecular Genetics, University of Virginia, Charlottesville, VA 22903, USA

## Abstract

**Summary:**

Nascent transcript measurements derived from run-on sequencing experiments are critical for the investigation of transcriptional mechanisms and regulatory networks. However, conventional mRNA gene annotations significantly differ from the boundaries of primary transcripts. New primary transcript annotations are needed to accurately interpret run-on data. We developed the primaryTranscriptAnnotation R package to infer the transcriptional start and termination sites of primary transcripts from genomic run-on data. We then used these inferred coordinates to annotate transcriptional units identified *de novo*. This package provides the novel utility to integrate data-driven primary transcript annotations with transcriptional unit coordinates identified in an unbiased manner. Highlighting the importance of using accurate primary transcript coordinates, we demonstrate that this new methodology increases the detection of differentially expressed transcripts and provides more accurate quantification of RNA polymerase pause indices.

**Availability and implementation:**

https://github.com/WarrenDavidAnderson/genomicsRpackage/tree/master/primaryTranscriptAnnotation.

**Supplementary information:**

Supplementary data are available at *Bioinformatics* online.

## 1 Introduction

Quantification of nascent transcription is critical for resolving temporal patterns of gene regulation and defining gene regulatory networks. Genome-wide nascent run-on sequencing methods are commonly used to quantify nascent transcription (Core *et al*., 2008; Kwak *et al*., 2013). Existing gene annotations are inadequate for both quantifying nascent transcripts and determining the RNA polymerase location relative to gene features. Analyses of run-on data indicate that annotated transcription start sites (TSSs) are often inaccurate. Moreover, transcription extends beyond the 3′ polyadenylation region (Proudfoot, 2016), thereby rendering transcription termination sites (TTSs) distinct from annotated mRNA ends. Identification of more accurate TSSs and TTSs for primary transcripts is important for accurate transcript quantification from run-on data. 

We present the R package primaryTranscriptAnnotation to directly infer TSSs and TTSs of annotated genes. This package also expands upon existing *de novo* transcription unit (TU) identification tools (Chae *et al.*, 2015; Heinz *et al.*, 2010) by facilitating the assignment of gene identifiers to the identified TUs. Our improved annotations increase the detection of differential transcript expression and quantification of RNA polymerase pausing. This package improves precision in analyses of critical phenomena related to transcriptional regulation and can be easily incorporated into standard genomic run-on analysis workflows.

## 2 Description

**Fig. 1.**
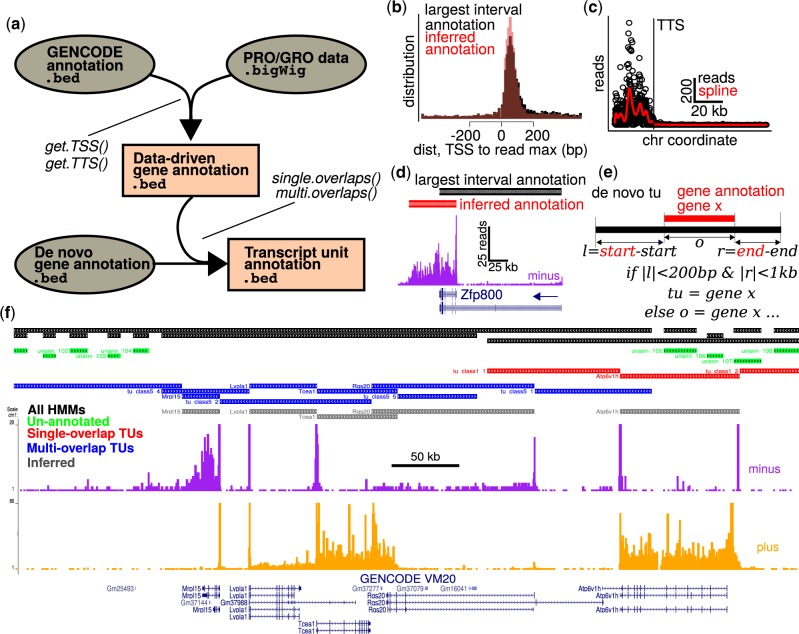
The primaryTranscriptAnnotation package accurately annotates gene features and assigns gene names to transcriptional units. (**a**) Aligned run-on data and gene annotations are inputs to redefine gene annotations. *De novo*-identified TUs can be assigned gene identifiers using refined gene annotations. (**b**) Promoter-proximal paused RNA polymerases are more constrained near the refined TSSs in comparison to conventional annotations. (**c**) TTS inference involves (i) detection of read density peaks in the 3′ end of the gene and (ii) determining the genomic position when the read density decays toward zero. (**d**) These methods generate an improved *Zfp800* annotation. (**e**) Annotation of *de novo*-defined TUs with gene identifiers is based upon the degree of overlap. (**f**) This approach produces gene boundaries with improved accuracy

The focus of this software package is to identify the coordinates of nascent transcripts corresponding to annotated genes. This tool provides a useful supplement to other tools used for nascent transcript analysis. For instance, dREG can be used to infer the coordinates of active transcription regulatory elements, such as enhancers, from nascent transcript data (Wang *et al.*, 2019). We distinguish two related tasks performed by our package: (i) integration of run-on data and existing gene annotations to refine estimates of TSSs and TTSs and (ii) combining the results of the first task with the results of an unsupervised TU identification method to annotate the TUs. We accept the data-driven annotations from (i) as a ‘ground truth’ and we use these coordinates to segment and assign identifiers to the *de novo* TUs (Fig. 1a). We demonstrate the package functions using PRO-seq data from adipogenesis time-series experiments. Extensive implementation details are provided with the publicly available R package.

### 2.1 Data-driven gene annotation and annotation of *de novo* transcriptional units

Curated gene annotations are a reference point for inferring TSSs and TTSs. We specifically focus on identifying the most prominent annotated TSSs, whereas novel TSSs can be identified using experimental methods such as 5′ GRO-seq, PRO-cap, and Start-seq (Mahat *et al.*, 2016; Scruggs *et al.*, 2015). To infer TSSs, we considered all first exons of each gene isoform. We defined the TSS as the 5′ end of the annotated first exon that contains the maximal read density within a specified downstream range. Such regions of peak read density exist at RNA polymerase ‘pause sites’ (Kwak *et al.*, 2013). To evaluate the performance of our TSS identification method, we compared our inferences to conventional ‘largest interval’ annotations. We defined the largest interval for each gene by taking the most upstream start coordinate and most downstream end coordinate from the curated annotation. Figure 1b and Supplementary Figure S1 show the distance between the gene start coordinate and the nearest read peak within 500 bases. Consistent with paused RNA polymerase accumulation in close proximity and downstream of transcription initiation sites, the distribution of peak RNA polymerase densities is more focused immediately downstream for the inferred TSS annotation as compared to the ‘largest interval’ annotation (Fig. 1b).

To infer TTSs, we examined evidence of transcriptional termination in regions extending from a 3′ interval of the gene to a selected number of base pairs downstream of the most distal annotated gene end (Supplementary Fig. S2). First, we defined a search region for identifying read density peaks corresponding to elevated polymerase density at gene ends (Supplementary Fig. S2). Elevated polymerase density at the gene ends occurs because transcription rates are attenuated there (Fong *et al.*, 2015). We defined the TTSs by binning the search regions, counting reads within the bins, fitting smooth spline curves to the binned counts, identifying peaks in the curves, and detecting points at which the curves decay from the peak toward zero (Fig. 1c and Supplementary Fig. S3). Results of our TTS identification procedures show that data-driven annotations detect the well-described phenomenon in which RNA polymerases transcribe beyond the polyadenylation and cleavage site (Fig. 1d and Supplementary Fig. S4). We note that instances of bidirectionally transcribed regulatory elements near the 3′ gene end could contribute to TTS identification (Supplementary Fig. S2). We estimated the upper bound on the prevalence of such occurrences and found that TTS identification could only be affected by bidirectional transcripts for up to 1.9% of the genes in our analysis (Supplementary Fig. S4e), consistent with the robustness of our approach.

While data-driven gene coordinates provide an improvement over conventional annotations, it can be advantageous to analyze run-on data in the context of TUs identified in an unbiased manner (Chae *et al.*, 2015). Given both *de novo* TUs and a trusted gene annotation, primaryTranscriptAnnotation combines these information sources to annotate the TUs so that TUs overlapping genes are assigned conventional gene names (Fig. 1e and Supplementary Figs S5 and S6). TUs that do not overlap any genes are marked as unannotated. Figure 1f shows PRO-seq reads along with *de novo* annotations derived from groHMM (black), TSS/TTS inference annotations (grey), and annotations based on combining the results of groHMM and TSS/TTS inference (green, red and blue). Combining the data-driven gene annotation and TU annotation methods provides more accurate transcript boundaries and retains gene identifier information to be used in downstream applications.

### 2.2 Improved detection of gene expression changes and RNA polymerase pausing

We annotated PRO-seq data with both ‘largest interval’ gene coordinates and inferred primary transcript coordinates (e.g. see Fig. 1d and Supplementary Fig. S7). We examined differential expression by applying a likelihood ratio test to our adipogenesis time-series run-on data. Our results demonstrate that our inferred annotations result in enhanced detection of differential expression (Supplementary Results and Fig. S8).

Promoter-proximal polymerase pausing has been implicated in numerous biological functions (Adelman and Lis, 2012; Duarte *et al.*, 2016). To determine if our inferred annotations confer an improvement for detecting RNA polymerase pausing, we computed ‘pause indices’ (Min *et al.*, 2011) based on data from inferred coordinates and largest interval annotations. Our methods enhance the quantification of pausing at a genome-wide scale (Supplementary Fig. S9).

## 3 Discussion

We describe the primaryTranscriptAnnotation package and illustrate its utility. The package requires minimal dependencies and is easy to use. primaryTranscriptAnnotation will be generally useful for investigations into the mechanisms of transcription.

## Data availability

Raw sequencing files and processed *bigWig* files are available from GEO accession record GSE133147.

## Supplementary Material

btaa011_Supplementary_Data
